# Outcomes of Pin and Plaster Versus Locking Plate in Distal Radius Intraarticular Fractures

**DOI:** 10.5812/traumamon.7951

**Published:** 2013-01-15

**Authors:** Mahmoud Bahari-Kashani, Mohammad Hosein Taraz-Jamshidy, Hassan Rahimi, Hami Ashraf, Masoud Mirkazemy, Amirreza Fatehi, Mariam Asadian, Jafar Rezazade

**Affiliations:** 1 Mashhad Orthopedic and Trauma Research Center, Faculty of Medicine, Mashhad University of Medical Sciences, Mashhad, IR Iran; 2 Department of Orthopedic Surgery, Faculty of Medicine, Mashhad University of Medical Sciences, Mashhad, IR Iran

**Keywords:** Radius Fracture, Internal Fixators, Treatment

## Abstract

**Background:**

Distal radius fractures are among the most prevalent fractures predictive of probable occurrence of other osteoporotic fractures. They are treated via a variety of methods, but the best treatment has not been defined yet.

**Objectives:**

This study was performed to compare the results of open reduction and internal fixation with locking plates versus the pin and plaster method.

**Materials and Methods:**

In this prospective study, 114 patients aged 40 to 60 years with Fernandez type III fracture referring to Imam-Reza and Mehr hospitals of Mashhad from 2009 to 2011, were selected randomly; after obtaining informed consent, they were treated with pin and plaster fixation (n = 57) or internal fixation with the volar locking plate (n = 57). They were compared at the one year follow up. Demographic features and standard radiographic indices were recorded and MAYO, DASH and SF - 36 tests were performed. Data was analyzed by SPSS software version 13, with descriptive indices, Mann-Whitney and Chi-square tests.

**Results:**

SF-36 test demonstrated a better general health (P < 0.001), mental health (P = 0.006), physical functioning (P < 0.001), social functioning (P < 0.001) and energy/fatigue (P < 0.001) in LCP group. However, pain (P = 0.647) was not significantly different between the groups. Physical limitation (P < 0.001) and emotional limitation (P < 0.001) were greater in the pin and plaster group. Also, in the LCP group mean MAYO score (P < 0.001) was more than pin and plaster group. Mean DASH score was not different between the groups (P = 0.218). The rate of acceptable results of radiographic indices (P < 0.001), grip strength (P < 0.001) and range of motion in supination-pronation (P < 0.001) in LCP method were better than the pin and plaster method.

**Conclusions:**

In treatment of intra-articular distal radius fractures in middle-aged patients internal fixation with locking plates may be prefered to pin and plaster as the treatment of choice.

## 1. Background

Distal radius fractures are the most prevalent osteoporotic fractures accounting for 16-17 percent of fractures (1, 2). These fractures, which mostly occur due to falling on open hand in elderly osteoporotic females, could predict the other probable osteoporotic fractures like pelvic fractures. There are several types of classifications. AO classification categorizes distal radius fractures into 3 groups, based on whether articular surface is involved or not (3). However, Fernandez categorizes distal radius fractures into five groups based on pathologic mechanism and the number of fractured pieces, and demonstrates the prognosis of the fracture perfectly. Types I, II, III and IV are due to bending, shearing, compressing, avulsion and high-velocity forces, respectively (3, 4). These fractures are accompanied with some complications such as median nerve damage (5), carpal tunnel syndrome (2), malunion (6), nonunion (2), strength loss, impaired forearm rotation, increase in transported force to the ulna and osteoarthritis (7, 8).

The best functional results, articular surface symmetry, and prevention of osteoarthritis in future, are the goals of treatment of distal radius intra-articular fractures (7). The first step in treatment is exact anatomic reduction (9, 10). We have to keep a balance between obtaining anatomic reduction, stable fixation, minimizing soft tissue damage and rapid movement for recovery in our selection of treatment options. Radiographic features for acceptable reduction of the distal radius consist of radial shortening less than 5mm, radial inclination more than 15mm, palmar tilt between 15 dorsal and 20 volar and articular surface step ≤ 2mm (2, 11). Treatment method must be determined by the fracture pattern, amount of displacement, stability of segments and articular surfaces, age and physical requirements of patients (12).

Different therapeutic methods have been proposed for these fractures; each have their own specific advantages and disadvantages. Pin and plaster is simple and common, but there are complications such as pin loosening, reduction failure, bone fracture at the site of the pin and infection (11). Green has shown acceptable results of pin and plaster treatment in 86% of distal radius intra-articular fractures in 75 patients (13). However, studies have demonstrated that plaster fixation often cannot preserve reduction and modify the length (14). In these cases, reduction will normally fail two weeks after plaster reduction (15). Spira reported unsuccessful results in 42% of intra-articular factures treated with plaster as well (16).

Open reduction and internal fixation (ORIF) has some advantages such as increased stability and rapid return of movement in unstable and intra-articular distal radius fractures. ORIF with LCP has good to perfect radiographic and functional results in comminuted intra-articular distal radius fractures and minimizes the number of unacceptable results (17, 18). The complications are surgical trauma, devascularization of segments, wrist stiffness, tendon irritation or rupture and the need for plate removal. In addition, this invasive method cannot be performed everywhere (19-23). Regarding to common use of pin and plaster in distal radius fracture in our country and lack of research on comparison of results of closed reduction and the pin and plaster method with open reduction and fixation with LCP in Iran, we decided to assess the results of these two therapeutic methods in 40-60 year-old patients with intra-articular distal radius fractures.

## 2.Objectives

This study was undertaken to compare the results of open reduction and internal fixation with locking plates to the pin and plaster method.

## 3. Materials and Methods

In this prospective study, 114 patients aged 40 to 60 years with Fernandez type III fracture referring to Imam-Reza and Mehr hospitals of Mashhad from 2009 to 2011 were treated with either pin and plaster fixation (n = 57) or internal fixation with volar locking plates (n = 57); they were compared after one year. The selection was randomized after obtaining informed consent. Exclusion criteria included specific diseases (malignancy, upper limb vascular disorder, hyperparathyroidism, multiple trauma, osteoarthritis, and rheumatoid arthritis), pathologic fracture, open fracture, concomitant fracture of the carpal bones and distal of ulna and history of ipsilateral distal radius fracture.

Demographic features were recorded, patients were examined and radiographs were taken one year after treatment. Grip strength was measured by means of mercury barometer. When the cuff of the mercury barometer was inflated to a fixed number, the patient was asked to compress the cuff until mercury level rises. The numbers were recorded for both hands and grip strength was calculated in percentages. Results of treatment were evaluated by means of three tests, MAYO, DASH, and SF-36. MAYO test (MAYO wrist score) is answered on a scale from 0 to 100 and consists of 4 parts, including pain (0 - 25), range of motion (0 - 25), grip length (0 - 25), and function (0-25). A score of 100 shows normal function (24).

The DASH test includes 30 questions; 21 questions evaluate the ability of doing special funtions and 9 questions evaluate the symptoms of patients with musculoskeletal problems of he upper limb. This test has a scale from 0 to 100 as well. Validity, reliability, internal consistency of the DASH test is high. Its Cronbach's alpha in English and Persian is 98 and 96, respectively (25, 26).

The SF-36 test is a 36-question test which measures physical and psychological health via 8 scales. They consist of physical function, physical limitation, pain, general health, energy / fatigue, social function, emotional function and mental health. This test is able to measure the quality of life, the load of disease and shows whether the treatment is cost-benefiting or not. The scores are added for each scale and converted to a 0-100 range. Reliability of this test is 80 to 85 in the English version. The median internal reliability of 8 items is 86 in the Persian version. The correlation between results of these 8 parts is important (27). Data was analyzed by SPSS software version 13. Median and standard deviation indices, scales and algorithms were used for descriptive statistics. The Mann-Whitney test was used for comparison of quantitative variables and Chi-square test was used for comparison of qualitative variables.

## 4. Results

There were 21 women and 36 men in the pin and plaster group and 17 women and 40 men in the locking plate group; they were assessed and sex distribution did not have significant difference in either group. The median age in pin and plaster and locking plate group was 41.7 and 42.4 years respectively. The most frequent mechanism of trauma was accidents in both groups. In all patients, fracture was an acute presentation, and they were operated within the first 24 to 48 hours. General health, mental health, physical functioning, social functioning and energy were better in the locking plate group. Pain did not have significant difference in either group, but physical and emotional problems were more in pin and plate group. Also, the median DASH score did not have significant difference in either group, but the median MAYO score was significantly higher in the locking plate group.

Chi-square test demonstrated that the number of acceptable cases of articular surface step, volar tilt, ulnar variance and radial inclination in the locking plate group was significantly greater. Also, the number of high-grade osteoarthritis was less in the locking plate group. Other complications include one case of infection at the ulnar pin site in the and plaster group, which responded to debridement and antibiotic therapy. Also, two cases of extensor tendon irritation due to exiting long screws dorsally and one case of EPL tendon rupture in the volar locking plate group.

## 5. Discussion

Distal radius fractures are the most common osteoporotic fractures in the elderly (2). This fracture is more common with predisposing factors such as osteoporosis, loss of balance, and decrease in visual aquity. Several studies have been performed to determine the best treatment based on the articular surface involvement and the number of frawgments. In this study, like some of previous studies (28), age and sex distribution did not have significant difference in the groups and these two variables have been controlled. The most frequent mechanism of trauma in both groups was car accidents. It is obvious that distal radius fracture in dominant hand will result in more severe functional problems (29). We had the same number of dominant hand involvement in both groups and this variable had been controlled as well (Table 1).

**Table1.** Demographic Information

	Pin & Plaster	Locking Plate	P- value
**Age**	41.7 ±1.7	42.4 ± 2.5	0.319
**Sex**			
Male	36 (%63.2)	40 (%70.2)	0.551
Female	21 (%36.8)	17 (%29.8)	
**Trauma mechanism**			
Falling	25 (%43.9)	21 (%36.8)	0.001
Motorcycle Accident	24 (%42.1)	28 (%49.1)	
Occupational Accident	8 (%14)	8 (%14)	
**Hand involved**			
Dominant	33 (%57.9)	28 (%49.1)	0.453
Non-Dominant	24 (%42.1)	29 (%50.9)	

In this study, SF-36 test showed better general and psychological health in the locking plate group as was physical function and energy; but there was no significant difference in pain between the groups. Physical and psychological problems were seen more in the pin and plaster group. Our results agreed with the results of previous studies (Table 2). In studies of Phadnis (28), Kwan (29), and Arora (30), all of which used ORIF, the median DASH score was less than this study. Kilic used Q-DASH test and its median score was calculated 8.3; this is less than the median DASH score of this study (31). Arora found no differance in ORIF vs. Simple casting DASH score (32). Wright stated that there was no significant differance in the DASH score of ORIF and external fixator (33). But Rizzo found the DASH score lower in ORIF vs. external fixator (34). This difference gradually decreases and there is no significant difference after a year (35). In the study of Phandis, the median MAYO score was greater than our study (90 versus 75.7)(28). In this study, the number of acceptable cases of articular surface step, palmar tilt, ulnar variance, radial inclination, the mean grip strength and the mean ROM of supination-pronation was better and high-grade osteoarthritis was less in the locking plate method, which shows better short-term and long-term results. (Table 3)

**Table 2.** The Mean and SD of SF-36, DASH and MAYO Scores in Two Treatment Groups

Test	Pin & Plaster		Locking Plate		P value
	Mean	SD	Mean	SD	
**SF-36**	42.1	22.3	66.5	27.4	0.001
Physical Functioning	54.8	15.9	27.6	31.9	0.001
Physical Role Limitation	54.3	16.2	28.6	26.2	0.001
Emotional Role Limitation	38.4	13.4	53.5	22.2	0.001
Energy/Fatigue	58.3	16.3	66.4	17.4	0.006
Mental Health	56.5	20.2	76.5	24.5	0.001
Social Functioning	56.0	14.3	52.64	16.3	0.647
Pain	54.3	7.7	62.8	14.1	0.001
General Health					
**DASHScore**	27.9	16.4	24.5	12.9	0.218
**MAYOScore**	60.7	11.3	75.2	19.5	0.001

**Table 3.** Range of Motion and Standard Radiographic Indexes in Both Treatment Groups

Variable	Group		P value
	Locking Plate	Pin & Plaster	
**Step**			0.001^a^
< 2mm	49 (%86)	29 (%50.9)	
> 2mm	8 (%14)	28 (%49.1)	
**Tilt**			0.001^a^
Non-Acceptable	0	12 (%21.0)	
Acceptable	57 (%100)	45 (%78.9)	
**Ulnar Variance**			0.001^a^
< 5mm	0	28 (1/49%)	
> 5mm	57 (%100)	29 (%50.9)	
**Inclination**			0.001^a^
Acceptable	0	24 (%42.1)	
Non-Acceptable	57 (%100)	33 (%57.9)	
**Range of Motion**			
**Flexion-Extension**	87.8 ± 8.5	87.1 ± 6.1	0.875^b^
**Supination-Pronation**	91.8 ± 7.1	76.5 ± 7.7	0.001^b^
**Grip Power**	90.1 ± 7.0	81.5 ± 8.8	0.001^b^

^a^ K2 Test

^b^ Mann-Whitney Test

In our study, radiographic results were significantly better in the locking plate group. In Kwan study 96-98% of patients had good to perfect results (29).

In another study, 88% of cases had good to perfect results (36). Arora reported that use of ORIF method in individuals aged more than 70 resulted in better radiographic results and less deformity (32). In a study by Lee, 40% of patients were completely pain free after ORIF treatment (37). Arora stated 71% were pain free (38). In our study, SF-36 test demonstrated that pain is the same in both methods of internal fixation with LCP and pin and plaster. In study of Arora, overall prevalence of complications was reported 27% and the most frequent complication was irritation and rupture of flexor and extensor tendons (30). In his study, prevalence of irritation and rupture of extensor tendons were 3.5% and 1.7% respectively. Several factors have role in selection of the therapeutic options in comminuted and intra-articular distal radius fractures. Daily requirements of patients have critical importance. In USA, selection of therapeutic option depends on age, location and insurance condition (38). Use of locking plates results in a perfect stable fracture reduction in osteoporotic bones. Although some studies have shown higher complications with locking plates it is the standard surgical treatment for intra-articular distal radius fractures in USA and Europe (30, 39). The use of locking plate in intra-articular distal radius fractures (Fernandez type III) in the elderly may be advantageous.
